# Parents’ and Teachers’ Perspectives on Children’s Socio-Emotional Well-Being During Transition from Home to Kindergarten

**DOI:** 10.3390/children12091145

**Published:** 2025-08-28

**Authors:** Sanja Tatalović Vorkapić, Tamara Komadina

**Affiliations:** 1 Faculty of Teacher Education, University of Rijeka, 51000 Rijeka, Croatia; 2 Kindergarten Fijolica, 51250 Novi Vinodolski, Croatia; tamara.komadina@dv-fijolica.hr

**Keywords:** children, parents, satisfaction with transition, social-emotional well-being, kindergarten teachers

## Abstract

Background: As the social-emotional well-being of children as a whole and specifically during the transition to kindergarten is of paramount importance, it is important to continuously research this topic using a multi-informant approach. Moreover, a further contribution of this study lies in addressing the substantial gap in the existing literature within this important field. Objectives: Starting from the Ecological-Dynamic Transition Model and the Positive Development and Resilience in Kindergarten (PERIK) Model, the main aim of this research was to analyze parents’ and teachers’ perceptions of children’s social-emotional well-being during the transition and adjustment, and the quality of transition and adjustment. Methods: The study was conducted on a sample of parents (N = 154; 147 mothers) and teachers from 4 kindergartens (N = 12, all female) as raters of children’s (N = 202; 82 girls) social-emotional well-being, using PERIK scale and four questions on the quality of transition. Results: All PERIK-dimensions were rated as elevated based on parents’ ratings and moderate based on teachers’ ratings. Ratings of difficulties during transition decreased, and satisfaction with transition and adjustment and cooperation between parents and caregivers during transition increased (teachers’ ratings were lower than parents’ ratings). The average duration of adjustment in kindergarten was three weeks. Correlation analyses showed the expected significant correlations between the PERIK dimensions and the quality of transitions and adjustment of children. Inter-rater agreement analyses showed the effect sizes were predominantly large and poor to medium agreement between parent and teacher ratings was determined. Conclusions: Although the study found that there are significant differences in perceptions of the relationship between PERIK-dimensions and satisfaction with children’s transition between teachers and parents, which was expected due to the assessment of children in different contexts, it is important to consider them both in future research.

## 1. Introduction

For children at early and preschool age, the transition from the family home to daycare or kindergarten represents the first significant step into the world [1]. In order to ensure that this transition is as smooth and supportive as possible, for the child as well as for their family, and to facilitate the continuation of expected developmental and learning trajectories, thorough preparation for this upcoming phase is essential [2]. During early childhood, children feel safest in the arms of their parents. However, as they begin to spend most of their day in early childhood care and education institutions, this role of providing emotional security is gradually assumed by kindergarten teachers, and, over time, by peers within the kindergarten [3]. Most kindergartens organize parent orientation meetings before a child’s first day of attendance, during which detailed information is provided regarding institutional routines, expectations, and essential guidelines for the transition [4,5,6]. According to the Croatian early childhood education system, children are eligible to enter nursery programs upon reaching one year of age (in some institutions from as early as six months of age). Throughout the preschool period, children may enroll in programs at any point as decided by their parents. The first mandatory participation in the formal education system in Croatia occurs in the year preceding entry into primary school (typically at age six), when children are enrolled in the compulsory preschool program [7]. The child’s first day at daycare initiates the formation of relationships between teachers, parents, and children, based on mutual trust, collaboration, and support, which are the elements essential for a successful adaptation process [8].

This transition marks the beginning of the adaptation within the institution, which is often more challenging for younger children due to their increased developmental needs and other influencing factors [9,10]. While the education system is expected to ensure pedagogical continuity to support development and learning in early and preschool-aged children, thereby facilitating transitions and adaptation, this is not always achieved in practice [11]. As a result, a wide array of challenges may arise, affecting not only children but also their caregivers, family members, teachers, and professional associates. Accordingly, the adaptation process is defined as *an individual’s capacity to adjust to their environment or novel circumstances* [12] (p. 272). Importantly, the contemporary paradigm shifts the focus from *adaptation to* the institution to *adaptation within* the institution [13], highlighting the central role and responsibility of early childhood education institutions in facilitating high-quality transitions and child adjustment.

Educational transitions and adjustments are embedded in an already dynamic sequence of developmental changes that naturally occur throughout early childhood [14]. This period is thus marked by numerous anticipated changes, with a particular emphasis on ensuring the socio-emotional well-being of children. The National Curriculum for Early and Preschool Education in Croatia [15] identifies one of the main objectives of early childhood care and education as the support of children’s socio-emotional well-being, particularly during critical periods such as transitions. In this process, the roles of significant adults, especially teachers and parents, are paramount for ensuring the quality of transitions and subsequent adjustment [16]. Their perceptions of transition quality can serve as a foundation for the development of effective guidelines and support mechanisms. Therefore, it is essential that both teachers’ and parents’ evaluations are integrated into research on this topic. In light of these considerations, the present study focuses on evaluating the quality of transitions and adjustment processes for children entering kindergartens, with particular attention to their socio-emotional well-being. Furthermore, it aims to explore the relationship between transition and adjustment quality and six dimensions of children’s socio-emotional well-being, using a multi-informant approach which encompasses ratings from two perspectives: teachers’ and parents’.

### 1.1. An Ecological Perspective on Children’s Transitions from Home to Kindergarten and Their Adjustment in Kindergarten

A transition is a dynamic process of change that begins the moment children (and their families) move from one set of circumstances to another [17] (p. 3) and continues until the child becomes a well-adjusted member of their new environment [18]. Upon entering kindergarten, children are exposed to unfamiliar situations. As they begin constructing a new identity within this new context, they attempt to make sense of the new rules and routines while integrating their prior knowledge and experiences with the current setting [19]. The outcomes of such early experiences are both short- and long-term, as the strategies children develop to adapt in a new environment are often employed in future contexts. The effectiveness of these strategies is of paramount importance. Numerous studies have shown that positive experiences during these crucial educational transitions are predictive of future success in social, emotional, and academic domains [20,21,22].

As described in earlier studies [6,23,24], the theoretical foundation for understanding childhood transitions is based on Bronfenbrenner’s ecological systems theory, which views development as occurring within a series of nested systems: micro-, meso-, exo-, and macro-systems [25]. Additionally, the temporal aspect, shaped by the specific historical, cultural, and social context in which children grow up, can strongly influence the developmental paths of different cohorts. By viewing transitions as ecological events, current literature has developed a comprehensive ecological model for understanding key factors related to early childhood transitions. Rimm-Kaufman and Pianta [26] were among the first to see the transition to kindergarten as a period marked by significant developmental vulnerability, during which children experience numerous major changes that create new expectations, such as the ability to follow structured routines and work more independently. It is therefore not surprising that their research found that about 16% of children were reported by teachers to have experienced serious adjustment difficulties during the transition to kindergarten, with another third facing moderate challenges [27,28].

From an ecological perspective, the child’s move to kindergarten is best understood as the result of interactions between intra- (e.g., characteristics of the child, parents, family, classroom, and broader community) and inter-personal factors (e.g., child–teacher relationships, family–kindergarten connections, parent–teacher collaboration), both synchronically and diachronically [27]. While all these factors are important for a full understanding of the quality of transitions, the ecological model highlights that the main focus is on establishing relationships between the family environment and the kindergarten setting, with the child’s optimal development as the key goal [27]. The basis of this ecological transition model is deeply rooted in the work of Bronfenbrenner and Morris [29], who elaborated on the two-way influences between children and the contexts they inhabit.

This framework provides a strong basis for examining relevant research questions, especially those related to children’s perceived socio-emotional well-being during transitions and adjustment, as well as strategies to improve it [6,9,23,30,31,32]. Importantly, such research must consider the different perceptions of key stakeholders. Analyzing the transition from home to kindergarten helps researchers and practitioners think about how these contexts change over time and what these changes mean. Central to the transition is creating meaningful contact and overlap between the family and kindergarten systems, most notably, developing parent-teacher relationships and collaborative practices. These newly formed relationships are vital for children’s successful adjustment in kindergarten [33]. In this way, the ecological transition model acts as a valuable analytical tool, capable of capturing the complexity of diverse situations and perspectives that characterize children’s transitional experiences.

### 1.2. Children’s Socio-Emotional Well-Being During Transition and Adjustment: A Multi-Informant Approach

The concept of child well-being refers to the state of optimal psychological functioning and experience, and is widely recognized as a central indicator of the quality of an educational process [34]. Child well-being is a multidimensional construct encompassing physical, motor, cognitive, emotional, and social dimensions that focus not only on the child’s present but also on their future life outcomes [35]. Drawing from the theoretical model of socio-emotional well-being and resilience developed by Mayr and Ulich [36], this construct is understood as a multidimensional phenomenon with a significant influence on child development, framed within a positive developmental perspective. It is grounded in three theoretical foundations: children’s mental health, resilience, and school readiness. Within the domain of mental health, the model integrates three core concepts: life skills, adult conceptions of well-being (hedonic and eudaimonic; [37]), and Becker’s work on mental health [38,39]. Life skills [40,41] refer to competencies enabling children to interact effectively with others and manage challenges. According to hedonic and eudaimonic frameworks, high-quality mental health requires satisfaction of both subjective internal drives and objective environmental needs, often a complex balance to achieve. The third concept views mental health through physical well-being, high energy levels, and expansiveness, traits later reflected in model dimensions such as assertiveness and initiative. In terms of resilience, the model highlights children’s ability to cope effectively with life challenges and emerge without manifest or latent cognitive, emotional, or behavioral impairment, showing continued positive developmental progress despite adversity [42,43]. In educational contexts, school readiness is another key concept, defined by environmental demands and essential child competencies: regulatory abilities, impulse control, emotional regulation, and self-directed exploratory behavior [44].

Based on these conceptual foundations and empirical validation, Mayr and Ulich [45] developed six factors of socio-emotional well-being and resilience. Self-control and thoughtfulness refer to children’s ability to manage their behavior, show empathy, and care for others. Social competence and relationship skills encompass the ability to form and maintain positive social relationships, engage in play, initiate communication, and involve peers in shared activities. Task/Activity orientation captures the child’s capacity to take responsibility for planning and execution of developmentally appropriate tasks or activities. While originally termed “task orientation,” a Croatian validation study [46] suggested the term “activity orientation” is more appropriate in early childhood settings, where structured tasks are replaced by free and guided activities. Assertiveness describes children’s capacity to express their emotions, needs, intentions, and desires in a confident and appropriate verbal manner. Emotional stability and stress management combine the ability to maintain emotional balance in challenging situations with the ability to regulate emotional reactivity and find effective coping strategies for calming down. Curiosity and pleasure in exploration, as the name suggests, refer to children’s enthusiasm for learning, openness to new situations, and positive emotional engagement with the world around them. Building upon this theoretical model, Mayr and Ulich [45] developed a standardized measurement instrument, which was later adapted and validated for use in Croatia [46] and employed in the present research.

The multi-informant approach to scientific assessment is widely utilized in developmental research, especially in studies of child mental health and well-being [34]. To obtain a comprehensive understanding of a child’s functioning, it is common to use assessments from multiple informants, ideally including both parents, teachers, and, where possible, the children themselves [47,48]. Multi-informant reports are a hallmark of high-quality developmental research because they offer multiple perspectives across different contexts, such as home and kindergarten, thus providing a more holistic view of the child [49]. While parents are often the primary informants due to their intimate knowledge of the child, their assessments are typically limited to the home environment and may carry a high degree of subjectivity. In contrast, kindergarten teachers serve as secondary informants who offer critical insights into the child’s behavior in educational settings, often characterized by structured social interactions and peer dynamics. There are three key advantages of using teachers as informants: (a) familiarity with each child, developed through substantial time spent together in daily routines; (b) objectivity, fostered by professional training in child observation; and (c) access to comparative data, based on classroom peer groups, allowing for relative assessments of behavior.

Thus, whenever feasible, collecting data from multiple relevant sources is essential, especially when the findings inform early interventions or educational programming. Overreliance on a single informant can lead to poor decision-making in school contexts [50]. While both parents and teachers contribute valuable insights, each carries distinct biases and limitations in expertise. Teachers may lack in-depth knowledge of children’s mental health and well-being, despite having access to peer-based behavioral benchmarks [51]. Screening processes that rely solely on one type of informant, either parent or teacher, often result in misidentification of children with mental health difficulties [52,53]. A key question arises: What should be done once data are collected from multiple informants? De Los Reyes et al. [54,55] argue that discrepancies between informants should not be automatically treated as measurement error to be minimized. In fields such as education [48], such differences often reflect valid, context-specific insights and may be uniquely meaningful [56,57]. Attempting to suppress these differences can be counterproductive.

To design and implement effective educational programs that support children’s socio-emotional well-being during transitions and adjustment in kindergarten, it is essential to integrate multi-informant data, a methodological principle that underpins the present study.

## 2. Research Aim, Tasks, and Hypotheses

The main aim of this study was to examine the levels of transition and adjustment quality in kindergarten and children’s socio-emotional well-being using a multi-informant approach, their relationship, and the level of inter-rater agreement. This comprehensive research covers these tasks:

(1) To assess the levels of transition and adjustment quality in kindergarten (measured by four questions about difficulties, satisfaction, collaboration, and adjustment duration) and children’s socio-emotional well-being (measured by six dimensions) through multiple informants, and to compare mean scores between parents’ and teachers’ ratings;
(2) To evaluate inter-rater agreement in transition quality and children’s socio-emotional well-being using correlation and reliability analyses;
(3) To investigate the relationship between transition quality and children’s socio-emotional well-being, including examining differences in two sets of correlations based on parents’ and teachers’ ratings.

Based on established theoretical models and prior research findings, the following hypotheses are postulated:

(1) It is expected to determine moderate to high levels of transition quality and socio-emotional well-being in children during their move from home to kindergarten and their adjustment process, with notable differences between parents’ and teachers’ ratings;
(2) Small to moderate inter-rater agreement in assessing transition quality and socio-emotional well-being during this period is expected to be found;
(3) Medium to high positive correlations between these variables, indicating a significant relationship between higher transition quality and better socio-emotional well-being in children, with noticeable differences depending on whether ratings are from parents or teachers’ ratings are expected to be determined.

## 3. Materials and Methods

### 3.1. Participants

The study included a total of 202 children from 12 kindergarten groups located across four towns in Croatia: Novi Vinodolski, Bribir, Tribalj, and Crikvenica. Among the participating children, 82 were girls and 110 were boys, with a mean age of M = 4.17 years (SD = 1.67; range = 1–7 years). Of the total sample, three children were identified as having special needs, primarily related to autism spectrum disorders, and one child had been born prematurely. Twelve kindergarten teachers and 154 parents participated in the study. Each teacher assessed the children in her respective group, while each parent evaluated their child. The teachers had a mean age of M = 39.75 years (SD = 10.76; range, 24–59 years) and an average professional experience of M = 16.75 years (SD = 10.39; range, 3–38 years). Of the 154 parents who participated, seven were fathers. The parents’ average age was M = 34.90 years (SD = 5.11; range = 24–50 years). Regarding marital status, 121 parents were married, 29 were in cohabiting partnerships, one was single, one was widowed, and two were divorced. Participants’ demographics could be observed in Table 1.

### 3.2. Measures

To assess the quality of children’s transition and adjustment to kindergarten, four items were used: (1) the level of difficulties children experienced during the transition and adjustment period, (2) the level of satisfaction with the transition and adjustment process, (3) the perceived quality of cooperation between parents and teacher during the transition and adjustment, and (4) the duration of the adjustment period. The first three items were rated for each child by both teachers and parents using a 5-point Likert-type scale, while the fourth item was open-ended, requiring respondents to indicate the number of weeks the adjustment process lasted for each child. The rating scales used were as follows: (a) Level of difficulties during transition and adjustment—from 1 (*completely absent*) to 5 (*completely present*); (b) Level of satisfaction with transition and adjustment—from 1 (*unsatisfactory*) to 5 (*satisfactory*); and (c) Quality of parent-teacher cooperation—from 1 (*very low*) to 5 (*very high*). The fourth item, regarding adjustment duration, was open-ended, with both teachers and parents instructed to specify the number of weeks.

To measure children’s socio-emotional well-being and resilience, the study used the Positive Development and Resilience in Kindergarten (PERIK) scale [45], specifically its Croatian adaptation and validation [46]. Originally, the PERIK scale existed in both German and English versions and included six subscales, each consisting of six items, for a total of 36 items. During translation and cultural adaptation to Croatian, two items were added to each subscale, except for the “Establishing Contact and Social Skills” subscale, which remained unchanged, and the “Assertiveness” subscale, which had three additional items, bringing the total number of items to 45. Kindergarten teachers and parents rated each item using a 5-point Likert-type scale: (1 = NO—*Strongly disagree*, 2 = no—*Partially disagree*, 3 = maybe—*Neither agree nor disagree*, 4 = yes—*Partially agree*, 5 = YES—*Strongly agree*). The original validation study [45] reported high and satisfactory reliability for each subscale: Making Contact/Social Skills (α = 0.88), Self-Regulation and Thoughtfulness (α = 0.86), Self-Assertiveness (α = 0.81), Emotional Stability (α = 0.82), Pleasure in Exploration (α = 0.86) and Task/Activity Orientation (α = 0.85). These findings were confirmed in the Croatian validation study [46]: Making Contact/Social Skills (α = 0.92), Self-Regulation and Thoughtfulness (α = 0.92), Self-Assertiveness (α = 0.87), Emotional Stability (α = 0.85), Pleasure in Exploration (α = 0.92). Reliability analysis conducted in the present study supported these previous findings. The results are presented in Table 2.

### 3.3. Procedure

This study represents an integral component of a larger research project supported by the University of Rijeka, Croatia. Concurrently, this part of the research formed a part of the master’s thesis of one of the co-authors. Before data collection, the Faculty of Teacher Education at the University of Rijeka issued an official request for collaboration with kindergartens, with which previous cooperation had been established within the framework of the broader research project, specifically within the Primorje-Gorski Kotar County. Following informed consent from the principals of the contacted kindergartens, kindergarten teachers agreed to participate in the study. Notices about the research were posted on bulletin boards intended for parents, ensuring they were informed about key aspects of the study. As parents had already provided general consent at the beginning of the pedagogical year for the collection of developmental data on their children (which aligns with the objectives of this study), they were only asked to notify the teachers if they did not wish to participate in this specific research, given that both teachers and parents were expected to complete assessments. After obtaining consent and providing detailed assessment instructions, each educational group received a set of rating scales. Teachers conducted their assessments using a paper-and-pencil method throughout 7 to 10 days. Once completed, the researchers collected the filled-out scales. Teachers generated unique identification codes for each child and assigned these codes accordingly. They then communicated the relevant code to each child’s parent. In this way, only teachers had access to the actual identities of the children, ensuring that researchers remained blinded to the participants’ identities, thus preserving anonymity and confidentiality. Based on the suggestion of kindergarten staff, parents were provided with a digital version of the scales via a link to a Google Forms application. They completed the questionnaires online. The collected data were processed using JASP 0.17.2.1. and SPSS 29, employing statistical techniques such as descriptive analysis, correlation analysis, intraclass reliability analysis, and paired-sample difference testing, along with effect size estimations.

## 4. Results

### 4.1. Parents’ and Kindergarten Teachers’ Ratings of the Transition and Adjustment Quality and Children’s Socio-Emotional Well-Being

Table 2 presents the results of the descriptive analysis, including means, standard deviations, ranges, and Cronbach’s alpha reliability coefficients for four transition quality variables: difficulties during transition and adjustment; satisfaction with the transition and adjustment process; quality of parent–teacher cooperation during the transition; and duration of adjustment (in weeks); as well as six dimensions of children’s socio-emotional well-being: social skills, self-regulation, self-assertiveness, emotional stability, activity orientation, and pleasure in exploration, as evaluated by both parents and kindergarten teachers.

Parents generally reported few difficulties during the adjustment period (M = 2.38), a perception shared by kindergarten teachers (M = 2.43), who likewise assessed the adjustment process as largely smooth with a low level of difficulties. Based on the collected data, parents rated the transition process as highly satisfactory (M = 4.60), whereas teachers assessed it as moderately to highly satisfactory (M = 4.11). A key component contributing to a successful adjustment process is the quality of parent–teacher cooperation. In this regard, parents reported a high level of collaboration with teachers (M = 4.48). Similarly, teachers also perceived an elevated level of cooperation with parents, highlighting the mutual importance of this relationship (M = 4.19). Both parents (M = 3.24) and teachers (M = 2.56) estimated the duration of the adjustment process to vary considerably, ranging from a single day to as long as six months, with an estimated average of 3 weeks of adjustment duration.

Regarding the children’s socio-emotional well-being during the transition and adjustment period, the highest-rated dimension, according to both parents (M = 4.44) and teachers (M = 3.79), was pleasure in exploration, while emotional stability received the lowest ratings (parents: M = 3.74; teachers: M = 3.37). When analyzing the parental assessments separately, elevated levels were observed across all six socio-emotional dimensions, indicating a generally positive perception of children’s adjustment. Teacher evaluations revealed the same relative pattern among the dimensions; however, overall ratings were lower than those provided by parents. Specifically, parents reported elevated levels in all dimensions, while teachers reported elevated levels only in pleasure in exploration and self-regulation (other dimensions were rated by teachers as moderate).

### 4.2. Inter-Rater Agreement on the Transition and Adjustment Quality and Children’s Socio-Emotional Well-Being

The second stage of the analysis focused on examining differences between parent and teacher mean scores for each of the four transition quality variables and the six dimensions of children’s socio-emotional well-being. A series of one-way repeated measures analyses of variance (ANOVAs) were conducted for this purpose. In addition, effect sizes were calculated for each variable using partial eta squared (η^2^_p_), and these are presented in Table 3 alongside the corresponding ANOVA statistics (F and *p* values). To interpret the magnitude of effect sizes, the classification proposed by Cohen [58] was applied: η^2^_p_ = 0.01 indicates a small effect, η^2^_p_ = 0.06 a medium effect, and η^2^_p_ = 0.14 a large effect. The analysis revealed statistically significant differences in two of the four transition quality variables: satisfaction with the transition and adjustment process, and the quality of cooperation between parents and teachers.

Specifically, parents reported significantly higher levels of satisfaction with the transition process and perceived a higher quality of cooperation with teachers than teachers did. Moreover, for all six dimensions of socio-emotional well-being, parents consistently rated their children’s adjustment significantly more positively than teachers. The effect sizes for these differences were predominantly large, indicating a robust discrepancy in perception between the two groups. In contrast, the effect sizes for differences in the variables difficulties during transition and adjustment, and adjustment duration were small. The effect size for the quality of parent–teacher cooperation was medium to large, while the effect size for satisfaction with transition and adjustment was large. To assess the degree of agreement between parent and teacher evaluations, two types of analyses were performed: (a) Pearson’s and Spearman’s correlation coefficients, to examine the strength and direction of the relationships between raters’ scores on both the transition quality variables and socio-emotional well-being dimensions, and (b) intraclass correlation coefficients (ICCs), to measure the absolute level of agreement between raters. The results of these analyses are presented in Table 3.

For the four transition quality variables, all correlations between parent and teacher ratings were statistically significant, indicating a shared perception to some degree. However, for the socio-emotional well-being dimensions, the results were more nuanced. Significant correlations were found for social skills, self-regulation, self-assertiveness, and activity orientation, while the correlation for emotional stability was marginally significant. No significant correlation was observed for pleasure in exploration. ICCs were calculated using a two-way random-effects model based on absolute agreement. The interpretation of ICC values followed Cicchetti’s [59] guidelines: values below 0.40 indicate poor agreement, 0.40 to 0.59 fair agreement, 0.60 to 0.74 good agreement, and values above 0.75 excellent agreement. Based on these criteria, poor agreement between parent and teacher ratings was observed for the following variables: difficulties during transition and adjustment, satisfaction with transition and adjustment, quality of parent–teacher cooperation, self-assertiveness, emotional stability, activity orientation, and pleasure in exploration. Fair agreement was established for adjustment duration, social skills, and self-regulation.

### 4.3. Parents’ and Kindergarten Teachers’ Perspective on the Correlation Between the Transition and Adjustment Quality and Children’s Socio-Emotional Well-Being

Finally, to explore the relationship between quality transition variables and dimensions of socio-emotional well-being among children during transition and adjustment in kindergarten, two separate correlation analyses were run, based on parents’ and teachers’ ratings, and Spearman’s correlation coefficients (due to significant deviations regarding Skewness and Kurtosis) with significance probability flags as can be seen in Table 4.

The parents’ ratings (upper rows in the cells) showed no significant relations between the first three transitions’ quality variables and dimensions of socio-emotional well-being, except in the case of pleasure in exploration. They evaluated that with the greater pleasure in exploration in children, a higher level of satisfaction with the transition and adjustment will be present, similar to what kindergarten teachers evaluated. In contrast to that, based on kindergarten teachers’ ratings, several significant correlations were determined: greater difficulties during transition and adjustment were related with lower social skills, self-regulation, self-assertiveness, emotional stability and pleasure in exploration; greater satisfaction with transition and adjustment was related with higher level of social skills, self-regulation, emotional stability, activity orientation and pleasure in exploration; and greater collaboration with parents was related with all six dimensions of socio-emotional well-being of children during transition and adjustment. Regarding the variable of adjustment duration in kindergarten, longer adjustment was related to lower social skills, self-assertiveness, and activity orientation based on parents’ ratings. Based on teachers’ ratings, it was determined that longer adjustment was related to lower social skills, self-assertiveness, emotional stability, and pleasure in exploration.

To analyze the significance of the difference between two correlations, an online calculator created by Daniel Soper [60] was applied. By this, it was possible to explore whether two correlation coefficients are significantly different from each other, those from parents’ and teachers’ ratings, given the two correlation coefficients and their associated sample sizes. Calculations resulted in z-scores and their probability level. A probability value of less than 0.05 indicates that the two correlation coefficients are significantly different from each other. Therefore, analyzing the determined findings showed in Table 4, it could be seen that there are five significant differences between correlation coefficients regarding the connection between transition quality and socio-emotional well-being: parents and teachers. Significantly greater correlation coefficients are determined in teachers than in parents’ ratings in these relationships: difficulties during transition-self-assertiveness; satisfaction with transition and adjustment-emotional stability; quality of parent-teacher collaboration-social skills; quality of parent-teacher collaboration-self-assertiveness; quality of parent-teacher collaboration-emotional stability.

Found significant correlations between quality transition and socio-demographic variables showed that parents perceive greater collaboration with kindergarten teachers if they are parents of girls and younger children, and longer adjustment in younger children. Based on teachers’ ratings, the significant correlations are determined related to greater difficulties during transition and adjustment, and longer adjustment in kindergarten among younger children; and the greater satisfaction with the transition and adjustment if they were younger, regarding their age. Within this set of correlations, parents’ and teachers’ ratings significantly differed in two relationships: parents rated the relationship between younger children and higher quality of collaboration with teachers as significant, while teachers didn’t; and teachers rated the relationship between their greater age and less satisfaction with transition and adjustment of children as significant, while parents didn’t.

Finally, analyzing the determined correlation between socio-emotional well-being and socio-demographic variables, it was determined that girls and older children have greater self-regulation and self-assertiveness, while older children also show greater social skills, based on parents’ ratings. Based on the teachers’ ratings, more correlations were found to be significant: girls were rated as more self-regulated, emotionally stable, and activity oriented, while older children showed higher all sic dimensions of socio-emotional well-being during transition and adjustment. In addition, based on teachers’ ratings, it was found that the self-assertiveness of children was dependent on teachers’ age: older teachers rated children as more self-assertive. Mostly, parents’ and teachers’ ratings significantly differed in correlations between children’s age and four dimensions of socio-emotional well-being: self-assertiveness, emotional stability, activity orientation, and pleasure in exploration, within which, correlations were significantly higher based on teachers’ ratings. In addition, teachers rated the relationship between their greater age and children’s greater self-assertiveness as significant, while parents didn’t.

## 5. Discussion

### 5.1. Children’s Socio-Emotional Well-Being During Transition and Adjustment, and the Transition and Adjustment Quality: Parents’ and Kindergarten Teachers’ Perspectives

In general, the observed levels of transition quality from home to preschool and children’s adjustment within preschool settings revealed results consistent with prior expectations and previously established findings. Specifically, these included lower levels of difficulties experienced by children during the transition and adjustment processes, a high level of collaboration between parents and teachers, and very high ratings of satisfaction during the transition and adjustment. These results are comparable to those from the national five-year longitudinal study [9], which was conducted on a sample of 795 children aged 1 to 5 years. In that study, both teachers (N = 77) and parents (N = 247) assessed the quality of transition and adjustment using identical quality transition variables. When comparing teachers’ assessments, the present study indicated slightly higher levels of difficulties experienced by children (M = 2.81), suggesting a moderate level of challenge. Meanwhile, satisfaction with the transition (M = 4.22) and collaboration between parents and teachers (M = 3.92) were both rated as elevated. In terms of parental evaluations, the national study reported a higher level of perceived difficulty (M = 2.93), but equally high levels of satisfaction with the transition and adjustment process (M = 4.60) and with parent-teacher collaboration (M = 4.43). It may thus be concluded that the findings are highly similar and largely expected, given that the majority of children experience a low level of difficulty during transitions and adjustment. Consequently, this contributes to increased satisfaction with the transition itself as well as with the collaboration between parents and teachers [16,27,28]. In addition, similar findings have been reported in studies beyond the domestic context, based on the perspectives of kindergarten teachers, parents, and children. Various international empirical studies have demonstrated that the majority of children experience positive and successful transitions when adequate support for their socio-emotional well-being is provided, characterized by satisfactory adjustment to kindergartens and effective cooperation with parents [61,62,63,64].

The implications of these findings point to the need for future research focusing on the detailed collection of qualitative data regarding the types and frequencies of difficulties encountered by children during transitions and adjustment. Moreover, it is necessary to examine how these difficulties vary according to institutional characteristics and other potentially significant variables. Such insights could inform the development of targeted support programs for children and parents during the transition process, ultimately reducing difficulties and enhancing satisfaction with both the transition and parent-teacher collaboration.

It is essential to consider both parental and teacher assessments, as statistical analyses revealed that teachers rated satisfaction with the transition and collaboration significantly lower than parents did. Given the large effect sizes observed, these differences should not be overlooked. Future studies employing qualitative methodologies may help to uncover the underlying reasons for these divergent perceptions. Interestingly, assessments did not significantly differ in terms of the estimated duration of children’s adjustment in kindergarten, which was reported as approximately three weeks. This finding aligns with previous research conducted in Croatia, in which 34 teachers estimated the average adjustment period as one month (i.e., four weeks) [6]. Stojić and colleagues [65] suggest that the adjustment period can range from 10–15 days up to two months, emphasizing the highly individual nature of adjustment, which is influenced by numerous factors, including the child’s age, developmental stage, emotional and health status, and temperament.

Moderate to high levels of social-emotional well-being dimensions, as rated by teachers, largely confirm findings from previous validation studies [45,46] as well as from the national study [9]. However, parental assessments indicated even higher levels across all dimensions compared to teacher assessments, which is again consistent with findings from the national study based on parental data. Statistical analyses, including significance testing and effect size analysis, revealed substantial differences between parental and teacher evaluations of all dimensions of children’s social-emotional well-being, with large effect sizes. This suggests a fundamental divergence in how these two groups perceive children’s social-emotional well-being during transitions. Overall, parents consistently rated their children’s well-being during transition and adaptation significantly higher than teachers did. This result aligns with prior expectations given the greater subjectivity typically present in parental assessments [47].

### 5.2. The Transition and Adjustment Quality and Children’s Socio-Emotional Well-Being During Transition: What Is the Level of Inter-Rater Agreement?

Conducting two types of analyses, Pearson’s and Spearman’s correlation coefficients, and intraclass correlation coefficients (ICCs) to assess inter-rater agreement revealed notable discrepancies between parents and kindergarten teachers in their evaluations of transition and adjustment quality, as well as children’s socio-emotional well-being during this period. With regard to the strength and direction of relationships between raters’ scores on the key variables, significant positive correlations were found for all four transition quality variables and the dimensions of socio-emotional well-being, with the exception of pleasure in exploration. These findings are consistent with ICC results, which indicated mostly poor to fair agreement between raters concerning both transition quality and socio-emotional well-being. Although low inter-rater agreement could potentially be attributed to low reliability, this explanation does not hold in this case. While slightly lower for parents, both groups’ ratings demonstrated high and satisfactory levels of internal consistency. Therefore, the observed discrepancies likely stem from children’s situation-specific behaviors and the differing evaluative standards used by informants [47,55,57].

Interestingly, the variables adjustment duration, social skills, and self-regulation showed fair levels of inter-rater agreement. This raises an important question: Are these particular behaviors easier to observe and therefore more consistently rated, or do these variables reflect dimensions that are more reliably and validly measured, or possibly both? Notably, reliability levels for social skills and self-regulation were highest in teacher ratings. However, regardless of reliability, children’s behavior appears to be highly context-dependent, varying significantly between the home and kindergarten environments. This underscores the importance of employing a multi-informant approach in studies of children’s socio-emotional well-being during transition and adjustment periods to capture a more comprehensive and nuanced understanding. Although there are currently no published studies focusing specifically on inter-rater agreement in the assessment of children’s socio-emotional well-being during transition to kindergarten, related studies in the field of child mental health report similar discrepancies between parent and teacher ratings [47,48,56,57]. In light of these findings, future research should incorporate the child’s perspective alongside those of parents and teachers [66]. Moreover, it is essential to include an equal representation of mothers and fathers to allow for more detailed analysis of multi-informant data. Each informant provides unique and valuable insight that, when considered together, can lead to a richer and more accurate understanding of children’s developmental experiences.

### 5.3. Parents’ and Kindergarten Teachers’ Perspectives on the Correlation Between the Transition and Adjustment Quality and Children’s Socio-Emotional Well-Being

Finally, given that previous research phases identified significant differences in mean scores for transition quality and children’s socio-emotional well-being during the transition and adjustment period, accompanied by large effect sizes, statistically significant yet low correlations, and poor inter-rater agreement based on calculated intraclass correlation coefficients (ICCs), the findings regarding significant differences in correlation coefficients reflecting the relationship between transition quality and socio-emotional well-being are to be expected. Overall, based on teacher ratings, more consistent and statistically significant correlations were observed between transition quality and all six dimensions of socio-emotional well-being, compared to parent ratings. These findings indicate that higher levels of socio-emotional well-being are significantly associated with higher-quality transitions from home to kindergarten and more successful child adjustment within the kindergarten setting. These relationships are consistent with previous research [9,32,67].

The study found that children assessed as having higher levels of social skills, self-regulation, pleasure in exploration, assertiveness, emotional stability, and activity orientation experienced significantly fewer difficulties during the transition process, as well as greater satisfaction with the transition and higher quality of collaboration between parents and teachers. Consistent with previous research, this study confirms that children in early and preschool education exhibit higher levels of social-emotional well-being and resilience across all dimensions when the transition process is perceived as highly satisfactory, collaboration with parents is of higher quality, and the number of experienced difficulties is lower. This finding represents one of the key outcomes of this complex study, which is primarily focused on children’s well-being during transitions and adaptation in preschool and primary school settings.

Given the significance of the identified correlations, it is critically important to ensure a high quality of transitions and adjustment, as these have a direct effect on children’s social-emotional well-being. Although these correlations were less pronounced in parental assessments, significant differences were observed in the relationships between difficulties and assertiveness, satisfaction with the transition and emotional stability, as well as between the quality of parent-teacher collaboration and three dimensions of social-emotional well-being: social skills, assertiveness, and emotional stability. The significance of these associations, as perceived by teachers, may be explained by the unique context in which teachers interact directly with children during the transition and adjustment periods, an experience that is only partially accessible to parents, for instance, during joint parent–child visits to the kindergarten setting. It is important to acknowledge that even in such shared contexts, parents do not have full insight into their child’s behavior in the new environment, since children typically behave differently in kindergarten settings depending on whether a parent is present or not. Consequently, differing perceptions between parents and teachers regarding children’s social-emotional well-being during transition and adjustment are to be expected. Additionally, Lopez and Benner [68] and Fukkink et al. [69] emphasize that the support provided by teachers during transitions is significantly associated with children’s social-emotional well-being in kindergarten. Effective collaboration and mutual respect between teachers and parents facilitate easier adjustment for the child, foster a sense of security and trust, and promote a higher level of social-emotional well-being. This is further supported by the findings on the variable measuring the quality of teacher-parent collaboration and its relationship to children’s social-emotional well-being, where the association was significantly stronger from the teachers’ perspective.

Furthermore, notable differences were found between parent and teacher perceptions in terms of the quality of transition, social-emotional well-being, and socio-demographic variables. Original validation studies demonstrated the expected significant associations between children’s general social-emotional well-being and their gender and age [45,46], as well as the association between these same variables and well-being during transition and adjustment [9,32,67], based on teacher assessments, differing from parental perspectives. Regarding age-related correlations, it is developmentally expected that younger children would experience more difficulties and lower satisfaction during the transition and adjustment period, along with lower levels of social-emotional well-being. This is due to the developmental trajectory of social-emotional competencies, which are directly related to children’s ability to cope with and adjust in new situations [45]. Teachers, due to their professional knowledge of developmental milestones and their ability to observe and compare behaviors across different age groups, are more sensitive to age-related correlations. Most parents lack access to these two key points of reference, which fundamentally affects their baseline for evaluating such constructs. Interestingly, although gender was found to affect collaboration quality, where cooperation was perceived as more effective with girls (according to parents), and higher dimensions of social-emotional well-being were observed in girls, these findings remain an open question. While gender-related correlational differences were identified in this study, there were no statistically significant differences between parent and teacher assessments in this regard. Previous research on transitional periods indicates that gender strongly influences all aspects of childhood development, although the degree and specific nature of its influence on transitions vary across families, communities, and cultures [14,70]. Moreover, studies on gender differences in children’s academic and behavioral outcomes, as indicators of effective adjustment, consistently show that girls outperform boys [2,71]. Therefore, future research should continue to explore the effects of gender and age on children’s social-emotional well-being during transitions and adjustment periods.

## 6. Conclusions

The primary aim of this study was to examine the quality of transition and children’s socio-emotional well-being, their interrelationship, as well as the inter-rater agreement between parents and teachers. In summary, the findings confirmed previous research indicating moderate to elevated levels of children’s socio-emotional well-being during the transition and adjustment period, as well as overall high quality of these transitions in kindergarten settings. As expected, although there are notable similarities in parents’ and teachers’ perceptions of these important aspects of transition and adaptation, which can serve as a basis for creating guidelines to facilitate the process, the study revealed a small to fair level of inter-rater agreement regarding parents’ and teachers’ ratings of transition quality and children’s socio-emotional well-being during this period. The explanation offered by Fält et al. [47] is applied in this study as well: parents and teachers possess distinct characteristics that significantly determine their assessments. Moreover, their evaluations are also shaped by context-specific situational factors. Therefore, when designing educational guidelines for practice, such as those aimed at facilitating smooth transitions and ensuring high levels of children’s socio-emotional well-being, it is crucial to employ a multi-informant approach in studies assessing children’s behavior.

Despite the study’s significant contribution, it is important to address its limitations, particularly when designing future research. As the sample was not randomly selected and included a relatively small number of parents (predominantly mothers), replication with a larger, randomly selected sample of children, parents, and teachers is warranted. As previously suggested, future studies should aim to include both parents in the assessment process, thereby enabling an examination of inter-rater agreement between mothers and fathers. Furthermore, as certain variables were not controlled, such as early childhood educators’ level of education and prior experience with similar assessments, they should be considered in future studies. Additionally, given that transition quality in this study was measured using only four items, future research should employ comprehensive transition quality assessment scales that capture multiple dimensions of this construct, allowing for a more nuanced understanding of the transition process. To develop practical guidelines, it would also be beneficial to apply qualitative methodologies to explore the causes and types of difficulties children experience during transitions. In addition, it would be useful to explore possible predictors of children’s socio-emotional well-being during transition and adjustment, and to see whether there are any differences in perceived predictors by parents and teachers. Such an approach could increase both child and parent satisfaction and enhance children’s socio-emotional well-being. Taken together, the findings of this study have significant implications for the professional practice of kindergarten teachers. The results provide both parents and teachers with a deeper understanding of children’s socio-emotional well-being during transitions and highlight the value of employing a multi-informant approach. Importantly, this study represents a rare contribution within the Croatian context and, for the first time, offers a detailed analysis of both parents’ and teachers’ perceptions of the transition process.

## Figures and Tables

**Table 1:** Participants’ (teachers, parents, children) demographics.

	Kindergarten Teachers	Parents	Children
N	12	154	202
Gender (frequencies)			
Female	12	147	82
Male	0	7	110
Age			
Mean	39.75	34.90	4.17
SD	10.76	5.11	1.67
Range	24–59	24–50	1–7
Teachers’ professional experience			
Mean	16.75		
SD	10.39		
Range	3–38		
Parents’ marital status (frequencies)			
Married		121	
Cohabiting partnerships		29	
Single		1	
Widowed		1	
Divorced		2	

**Table 2:** Descriptive parameters (M = Mean, SD = Standard Deviation, Range, and Cronbach alpha α) for 4 transition quality variables and 6 dimensions of socio-emotional well-being of children rated by parents and kindergarten teachers.

		Parents				Kindergarten Teachers			
		M	SD	RANGE	α	M	SD	RANGE	α
Satisfaction with the quality of transition from home to kindergarten	Difficulties during transition and adjustment	2.38	1.16	1–5		2.43	1.15	1–5	
	Satisfaction with transition and adjustment	4.60	0.65	1–5		4.11	0.93	1–5	
	Quality of parent-teacher cooperation	4.48	0.80	1–5		4.19	0.76	2–5	
	Adjustment duration (in weeks)	3.24	4.04	0–24		2.56	5.54	0.2–24	
Socio-emotional well-being and resilience	Social skills	4.05	0.77	1.33–5.00	0.866	3.55	1.06	1.00–5.00	0.946
	Self-regulation	3.90	0.68	1.13–5.00	0.867	3.70	0.98	1.00–5.00	0.947
	Self-assertiveness	3.98	0.77	1.14–5.00	0.861	3.45	0.96	1.14–5.00	0.912
	Emotional stability	3.74	0.64	2.00–5.00	0.805	3.37	0.71	1.00–5.00	0.847
	Activity orientation	3.77	0.66	1.75–4.88	0.781	3.42	0.78	1.25–5.00	0.863
	Pleasure in exploration	4.44	0.60	1.00–5.00	0.884	3.79	0.83	1.00–5.00	0.923

**Table 3:** Mean differences (F, *p*), effect sizes (η^2^*_p_*), Pearson (r) and Spearman (rho) inter-rater correlations, and inter-rater agreement (ICC, lower and upper bound) for 4 transitions quality variables and 6 dimensions of socio-emotional well-being.

		ANOVA			Parents and Teachers	Inter-Rater Agreement 95% Confidence Interval		
		F	*p*	η^2^*_p_*	r (rho)	ICC	Lower Bound	Upper Bound
Satisfaction with the quality of transition from home to kindergarten	Difficulties during transition and adjustment	1.48	0.23	0.01	0.21 * (0.22 *)	0.21	0.09	0.33
	Satisfaction with transition and adjustment	22.51	**0.001**	0.17	0.24 ** (0.20 *)	0.25	0.12	0.36
	Quality of parent-teacher cooperation	9.29	**0.002**	0.08	0.25 ** (0.27 **)	0.25	0.13	0.36
	Adjustment duration (in weeks)	3.08	0.08	0.03	0.54 *** (0.27 **)	0.44	0.33	0.54
Socio-emotional well-being and resilience	Social skills	42.29	**0.000**	0.27	0.52 *** (0.48 ***)	0.52	0.42	0.60
	Self-regulation	9.61	**0.002**	0.08	0.46 *** (0.45 ***)	0.45	0.34	0.54
	Self-assertiveness	34.78	**0.000**	0.24	0.31 ** (0.30 **)	0.34	0.22	0.44
	Emotional stability	27.04	**0.000**	0.19	0.19 * (0.15)	0.20	0.07	0.32
	Activity orientation	23.69	**0.000**	0.18	0.34 ** (0.28 **)	0.36	0.25	0.47
	Pleasure in exploration	53.89	**0.000**	0.32	0.13 (−0.04)	0.13	0.01	0.25

* *p* < 0.05, ** *p* < 0.01, *** *p* < 0.001. Note: Bolded values are significant

**Table 4:** Spearman’s correlation coefficients and flagged probability for 4 transition quality variables, socio-demographic variables, and 6 dimensions of socio-emotional well-being of children based on parents’ (N = 145; upper row) and kindergarten teachers’ ratings (N = 202; lower row) with z-scores and probability levels for significance of difference testing (cells with significant z-scores are marked in grey colour).

	Difficulties During Transition and Adjustment		Satisfaction with Transition and Adjustment		Quality of Parent-Teacher Cooperation		Adjustment Duration (In Weeks)		Children’s Gender		Children’s Age		Parents’ or Teachers’ Age	
Social skills	−0.06 −0.23 ***	1.61 (0.11)	0.14 0.22 **	−0.77 (0.44)	0.09 0.29 ***	**−1.93** **(0.05)**	−0.27 *** −0.19 **	−0.78 (0.43)	0.05 0.09	0.37 (0.71)	0.28 *** 0.45 ***	−1.82 (0.07)	−0.03 0.05	−0.74 (0.46)
Self-regulation	−0.06 −0.20 **	1.32 (0.19)	0.09 0.18 *	−0.85 (0.40)	0.11 0.30 ***	−1.84 (0.06)	−0.07 −0.11	0.37 (0.71)	0.16 * 0.21 **	−0.48 (0.63)	0.32 *** 0.48 ***	−1.77 (0.08)	0.03 0.12	−0.84 (0.40)
Self-assertiveness	0.06 −0.17 *	**2.14** **(0.03)**	0.08 0.15	−0.66 (0.51)	−0.02 0.22 **	**−2.26** **(0.02)**	−0.16 * −0.17 *	0.10 (0.92)	0.16 * 0.07	0.84 (0.40)	0.30 *** 0.51 ***	**−2.34** **(0.02)**	−0.05 0.16 *	**−1.96** **(0.05)**
Emotional stability	−0.11 −0.28 ***	1.64 (0.10)	0.12 0.32 ***	**−1.95** **(0.05)**	0.08 0.32 ***	**−2.33** **(0.02)**	−0.13 −0.18 *	0.47 (0.64)	0.12 0.14 *	−0.19 (0.85)	0.05 0.26 ***	**−2.00** **(0.05)**	−0.02 0.01	−0.28 (0.78)
Activity orientation	−0.08 −0.14	0.56 (0.57)	0.15 0.17 *	−0.19 (0.85)	0.16 0.28 ***	−1.17 (0.24)	−0.18 * −0.05	−1.22 (0.22)	0.05 0.25 ***	−1.90 (0.06)	0.15 0.36 ***	**−2.08** **(0.04)**	−0.00 0.10	−0.93 (0.35)
Pleasure in exploration	−0.09 −0.14 *	0.47 (0.64)	0.19 * 0.27 ***	−0.78 (0.43)	0.08 0.26 ***	−1.72 (0.09)	−0.09 −0.14 *	0.47 (0.64)	0.04 0.12	−0.74 (0.46)	−0.09 0.29 ***	**−3.60** **(0.001)**	−0.09 0.03	−1.11 (0.27)
Children’s gender	0.01 0.06	−0.46 (0.64)	0.00 −0.03	−0.28 (0.78)	0.20 * 0.08	1.14 (0.26)	0.05 0.03	0.19 (0.85)						
Children’s age	−0.11 −0.24 ***	1.24 (0.21)	−0.14 −0.11	−0.28 (0.78)	−0.24 ** 0.05	**−2.73** **(0.01)**	−0.25 ** −0.28 ***	0.30 (0.76)						
Parents’ or teachers’ age	−0.06 −0.06	0.00 (1.00)	0.01 −0.23 **	**2.26** **(0.02)**	−0.04 0.03	−0.65 (0.52)	−0.05 −0.10	0.47 (0.64)						

* *p* < 0.05, ** *p* < 0.01, *** *p* < 0.001. Note: Bolded values are significant

## Data Availability

The data underlying this article cannot be shared publicly because of the privacy and confidentiality of the study participants, which are protected. The data of this study are available upon reasonable request from the corresponding author.
